# Polypharmacy and associated factors: a gender perspective in the elderly Spanish population (2011–2020)

**DOI:** 10.3389/fphar.2023.1189644

**Published:** 2023-04-21

**Authors:** Jesús Cebrino, Silvia Portero de la Cruz

**Affiliations:** ^1^ Department of Preventive Medicine and Public Health, University of Seville, Seville, Spain; ^2^ Department of Nursing, Pharmacology and Physiotherapy, University of Córdoba, Córdoba, Spain; ^3^ Research Group GC12 Clinical and Epidemiological Research in Primary Care, Instituto Maimónides de Investigación Biomédica de Córdoba (IMIBIC), Hospital Universitario Reina Sofía, Córdoba, Spain

**Keywords:** aged, gender perspective, pharmaceutical preparations, polypharmacy, trends

## Abstract

**Background:** Few studies have examined the epidemiology of polypharmacy in non-institutionalized elderly adults with regard to sex differences. This study aimed to identify the prevalence of polypharmacy among people ≥65 years old residing in Spain, analyze trends in that prevalence from 2011/12 to 2020, describe the use of the medicines involved and study the possible relationship between polypharmacy and certain sociodemographic, health-related variables, as well as the use of care services by sex.

**Methods:** A nationwide cross-sectional study with 21,841 non-institutionalized people ≥65 years old from the Spanish National Health Survey (2011/2012 and 2017) and the European Health Survey in Spain (2014 and 2020) was performed. We used descriptive statistics, performing two binary logistic regressions to determine the factors related to polypharmacy.

**Results:** The prevalence of polypharmacy was 23.2% (women: 28.1%, men: 17.2%; *p* < 0.001). The most commonly consumed medicines were analgesics and tranquillizers, relaxants or sleeping pills in elderly women, compared with antihypertensives, antacids and antiulcer drugs and statins for elderly men. In both sexs, the positive predictors of polypharmacy included average, poor and very poor self-perceived states of health, people with overweight and obesity, being severely/non-severely limited due to a health problem, having ≥ three chronic conditions, visits to the family doctor and hospitalization. Among elderly women, negative predictors were alcohol intake, whereas in elderly men positive predictors were being 75–84 years old, being current smokers and having 1, 2 chronic conditions.

**Conclusion:** Polypharmacy has a prevalence of 23.2%, with women accounting for 28.1% and men 17.2% of the total. Knowledge of positive and negative predictors of polypharmacy have important implications for public health efforts to develop or improve health guidelines and strategies for promoting the proper use of medication, particularly in the elderly population by sex.

## 1 Introduction

It is extremely challenging to describe the frequency and health consequences of polypharmacy in elderly people, since there is no consensus over one universal definition of polypharmacy (Sirois et al., 2019). In this context, some authors define “polypharmacy” as the precise number of multiple medicines taken by a patient, while others define it as optimizing the relevant medicines so that the patient takes the least number possible (Mortazavi et al., 2016). Nevertheless, most definitions of polypharmacy are numerical, and generally coincide in the concurrent use of ≥5 medicines (Payne, 2016; Masnoon et al., 2017; Rochon, 2022). Furthermore, most elderly people who use drugs are women (Venturini et al., 2011), besides the fact that due to their longer lifespan, women constitute the majority of long-term care residents (Rochon et al., 2021).

In the Spanish context, previous studies based on the Spanish National Health Survey (SNHS) or the European Health Interview Survey for Spain (EHIS) showed a prevalence of polypharmacy of 19.7% (SNHS 2006) and 24.5% (SNHS 2012) (Martin-Pérez et al., 2017). Similarly, Carmona-Torres et al. (Carmona-Torres et al., 2018) estimated a prevalence of 21.9% after analyzing jointly SNHS 2006, SNHS 2011/12, EHIS 2009, and EHIS 2014. In contrast, Gutiérrez-Valencia et al. (Gutiérrez-Valencia et al., 2019) obtained a prevalence of 27.3% using SNHS 2017. Finally, one European study showed a range of prevalence between 26.3% and 39.9% for Europe, with a figure of 31.6% for Spain (Midão et al., 2018).

Polypharmacy is influenced by the clinical guidelines for treating individual diseases, by which health professionals prescribe specific medicines for each disease separately (Christensen et al., 2019). However, women are less likely than men to receive and adhere to the medical treatment and monitoring recommended by clinical guidelines; however, women are also more likely than men to use one or more medications and, on average, more specialized medication than men (Manteuffel et al., 2014).

Among the many factors which predispose patients to polypharmacy are educational level, demographics or living in a nursing home (Khezrian et al., 2020), age, obesity, tobacco and alcohol consumption and economic conditions (Slater et al., 2018). Gender-related sociodemographic and health-related factors may also have a role to play (Jyrkkä et al., 2009; Nobili et al., 2011; Maher et al., 2014).

The study of polypharmacy continues to be crucial and constitutes a key opportunity for health professionals to develop guidelines to improve patient safety (Araújo et al., 2019). For all the above reasons, gaining greater knowledge about polypharmacy in elderly populations has been an ongoing, international concern (Rankin et al., 2018). Nevertheless, few studies have examined the epidemiology of polypharmacy in non-institutionalized elderly adults (Aparasu et al., 2005) with regard to sex differences (Lagerin et al., 2020). In this study, we therefore highlight the importance of exploring sex differences in this stage of life to improve our understanding of this significant issue, with the following aims: i) to study the prevalence of polypharmacy among people ≥65 years old residing in Spain, ii) to analyze trends in that prevalence from 2011/12 to 2020, iii) to describe the use of medicine, and iv) to identify the predictors of polypharmacy by sex.

## 2 Material and methods

### 2.1 Design, data source and participants

To conduct this nationwide cross-sectional study, we used secondary data from the personalized interviews of the SNHS 2011/12 (from July 2011 to July 2012) (Ministry of Health Social Services and Equality National Institute of Statistics, 2013), the SNHS 2017 (from October 2016 to October 2017) (Ministry of Health Consumer Affairs and Social Welfare National Institute of Statistics, 2017a), the EHIS 2014 (from January 2014 to January 2015) (Ministry of Health and Social Services and Equality National Institute of Statistics, 2015) and the EHIS 2020 (from July 2019 to July 2020) (Ministry of Health National Institute of Statistics, 2020a). The interviews were conducted with non-institutionalized members of the community living mainly in family homes (households) in Spain by the Ministry of Health, in partnership with the National Institute of Statistics. A three-stage probabilistic design was used, with data stratified by census areas (first stage), sections (second stage), and individuals (third stage). The team who administered the survey had previously been taught basic communication skills, associated processes and in particular, training in administering questionnaires. The participants were notified about the survey via letter, which explained the reasons for the survey, as well as the voluntary and anonymous nature of participation, and informed them that a suitably qualified interviewer would visit them. All the participants completed informed consent forms. More information about the methodology of the SNHS 2011/12 and 2017 and the EHIS 2014 and 2020 can be found elsewhere (Ministry of Health and Social Services and Equality National Institute of Statistics, 2013; Ministry of Health Social Services and Equality and National Institute of Statistics, 2015; Ministry of Health Consumer Affairs and Social Welfare National Institute of Statistics, 2017b; Ministry of Health National Institute of Statistics, 2020b).

The sample was representative of the elderly population (≥65 years old) residing in Spain and originally consisted of 26,604 participants (SNHS 2011/12: *n* = 5,896; EHIS 2014: *n* = 6,519; SNHS 2017: *n* = 7,022, and EHIS 2020: *n* = 7,167). However, due to a lack of data for some of the variables studied, 4,763 (17.90%) were excluded from the descriptive, bivariate, and multivariate statistical analyses (SNHS 2011/12: *n* = 1,549; EHIS 2014: *n* = 931; SNHS 2017: *n* = 1,023, and EHIS 2020: *n* = 1,260). Finally, the total sample numbered 21,841: 4,347 in SNHS 2011/12; 5,588 in EHIS 2014; 5,999 in SNHS 2017, and 5,907 in EHIS 2020.

### 2.2 Outcome measurements

The dependent variable was “polypharmacy”, which was assessed using an identical question in all the questionnaires: “From the following medications, which have you consumed in the last 2 weeks?”. Participants were classified as polypharmacy (yes/no) if they answered “yes” to the question about ≥5 different medicines: medicines for colds, flu, throat, bronchi; analgesics; medicines to lower a fever; restorative medicines, such as vitamins, minerals, tonics; laxatives; antibiotics; tranquillizers, relaxants, sleeping pills; allergy medication; diarrhea medication; medicines for rheumatism; heart medication; antihypertensives; antacids and antiulcer drugs; antidepressants, stimulants; weight loss medication; statins; diabetes medication; thyroid medication and other medication. As the study population were aged ≥65 years old, contraceptive pills and menopausal hormones were not considered. Although no consensus has yet been reached on the number of medicines that must be consumed to be included in the “polypharmacy” category, the threshold of ≥5 different medications was chosen because it has been used in recent studies conducted in various countries (Eiras et al., 2016; Lopes et al., 2016; Urfer et al., 2016) and is the most widely used formula (4).

### 2.3 Sociodemographic, health-related variables and use of clinical care services

The independent variables were classified into three groups: i) Sociodemographic variables, ii) health-related determinants and iii) use of clinical care services.

(i) Sociodemographic variables: Year of the surveys (2011/12, 2014, 2017, 2020); sex (women, men); age intervals (65–74 years, 75–84 years, ≥85 years); marital status (single, married, widowed, separated or divorced); educational level (without studies, primary, secondary or professional training, university); nationality (Spanish, foreigner); size of town (rural, urban); and social class (social classes I and II, social classes III and IV, social classes V and VI) (Domingo-Salvany et al., 2013).
(ii) Health-related variables: Self-perceived state of health (very good, good, average, bad, very bad); current smoker (yes, no); alcohol intake in the last year (yes, no); degree of limitation due to a health problem for at least 6 months (severely limited, limited but not severely, not at all limited); number of medical diagnoses of chronic conditions (none, 1–2, ≥3); and Body Mass Index (BMI) (underweight, normal weight, overweight, obese) (World Health Organization WHO, 2022).
(iii) Use of clinical care services: Number of visits to the family doctor in the preceding 4 weeks (yes, no) and number of hospitalizations in the past 12 months (yes, no).

### 2.4 Procedure and ethical considerations

The downloaded anonymized data is available to the general public via the National Institute of Statistics and the Ministry of Health websites (Ministry of Health Social Services and Equality National Institute of Statistics, 2013; Ministry of Health and Social Services and Equality National Institute of Statistics, 2015; Ministry of Health Consumer Affairs and Social Welfare National Institute of Statistics, 2017a; Ministry of Health National Institute of Statistics, 2020a). When using secondary data, approval by the Ethics Committee is not required, according to Spanish law.

### 2.5 Statistical analysis

A descriptive analysis was performed on the qualitative variables, using counts and percentages, and the quantitative variables, using arithmetic mean and standard deviation (SD). The Chi-square test was used for contingency tables, and Fisher’s exact test was used if the number of expected frequencies was greater than 5. For each sex, we performed a logistic regression model. We included all the variables whose univariate test showed a potential association with the dependent variable (*p* ≤ 0.15), and backward selection was used to eliminate non-significant variables based on the probability of the Wald statistic. Crude and adjusted Odds Ratios (OR) were calculated with 95% confidence intervals. The goodness of fit was verified using the Hosmer–Lemeshow test. A *p*-value ≤ 0.05 was considered to be significant. All data analyses were performed separately according to the sex (women, men). The IBM SPSS Statistical package version 26.0.0 (IBM Corp, Armonk, NY, United State) was used for the statistical analysis, which was licensed to the University of Seville (Spain).

## 3 Results

### 3.1 Comparison of women and men as regards sociodemographic, health-related variables and use of clinical care services

The records of 21,841 participants residing in Spain were analyzed, including 55.4% (*n* = 12,096) women and 44.6% (*n* = 9,745) men. Table 1 shows that the groups differ in all the study variables, except in nationality. For example, women lived more frequently in urban areas (*p* < 0.001) and had not consumed alcohol in the last year (*p* < 0.001) than men. In turn, men were more frequently classified as overweight (*p* < 0.001) and having ≥3 chronic conditions (*p* < 0.001) than women.

**TABLE 1:** Comparison of women and men as regards sociodemographic, health-related variables and use of clinical care services (N = 21,841).

Variables	Total	Women *n* = 12,096 (55.4%)	Men *n* = 9,745 (44.6%)	*p*-value
	N = 21,841 (100%)			
Age intervals				
65–74 years	11,133 (51.0)	5,855 (48.4)	5,278 (54.2)	
75–84 years	7,789 (35.7)	4,372 (36.1)	3,417 (35.1)	<0.001
≥85 years	2,919 (13.3)	1869 (15.5)	1,050 (10.7)	
Marital status				
Single	1834 (8.4)	890 (7.4)	944 (9.7)	
Married	11,781 (53.9)	4,986 (41.2)	6,795 (69.7)	<0.001
Widowed	7,276 (33.3)	5,762 (47.6)	1,514 (15.5)	
Separated or divorced	950 (4.4)	458 (3.8)	492 (5.1)	
Social class				
Social classes I and II	3,226 (14.8)	1,645 (13.6)	1,581 (16.2)	
Social classes III and IV	7,540 (34.5)	4,109 (34.0)	3,431 (35.2)	<0.001
Social classes V and VI	11,075 (50.7)	6,342 (52.4)	4,733 (48.6)	
Educational level				
Without studies	6,385 (29.2)	3,966 (32.8)	2,419 (24.8)	
Primary	7,730 (35.4)	4,376 (36.2)	3,354 (34.4)	<0.001
Secondary or PT	5,729 (26.2)	2,904 (24.0)	2,825 (29.0)	
University	1997 (9.2)	850 (7.0)	1,147 (11.8)	
Nationality				
Spanish	21,529 (98.6)	11,929 (98.6)	9,600 (98.5)	0.51
Foreigner	312 (1.4)	167 (1.4)	145 (1.5)	
Size of town				
Rural	5,720 (26.2)	2,899 (24.0)	2,821 (28.9.0)	<0.001
Urban	16,121 (73.8)	9,197 (76.0)	6,924 (71.1)	
Self-perceived state of health				
Very good	1,501 (6.9)	754 (6.2)	747 (7.7)	
Good	8,854 (40.5)	4,330 (35.8)	4,524 (46.4)	
Average	7,754 (35.5)	4,593 (38.0)	3,161 (32.4)	<0.001
Bad	2,907 (13.3)	1850 (15.3)	1,057 (10.9)	
Very bad	825 (3.8)	569 (4.7)	256 (2.6)	
Current smoker				
Yes	1941 (8.9)	622 (5.1)	1,319 (13.5)	<0.001
No	19,900 (91.1)	11,474 (94.9)	8,426 (86.5)	
Alcohol intake in the last year				
Yes	9,832 (45.0)	4,057 (33.5)	5,775 (59.3)	<0.001
No	12,009 (55.0)	8,039 (66.5)	3,970 (40.7)	
Body Mass Index				
Underweight	233 (1.1)	178 (1.5)	55 (0.6)	
Normal weight	6,693 (30.6)	4,099 (33.9)	2,594 (26.6)	<0.001
Overweight	9,920 (45.4)	4,897 (40.5)	5,023 (51.6)	
Obesity	4,995 (22.9)	2,922 (24.1)	2073 (21.2)	
Degree of limitation due to a health problem for at least 6 months				
Severely limited	2,234 (10.2)	1,471 (12.1)	763 (7.8)	
Limited but not severely	7,667 (35.1)	4,715 (39.0)	2,952 (30.3)	<0.001
Not at all limited	11,940 (54.7)	5,910 (48.9)	6,030 (61.9)	
Number of visits to the family doctor in the preceding 4 weeks				
0	13,291 (60.8)	7,257 (60.0)	6,034 (61.9)	
1	6,853 (31.4)	3,826 (31.6)	3,027 (31.1)	<0.001
≥2	1,697 (7.8)	1,013 (8.4)	684 (7.0)	
Number of hospitalizations during the past 12 months				
0	18,989 (86.9)	10,612 (87.7)	8,377 (86.0)	
1	2,205 (10.1)	1,140 (9.4)	1,065 (10.9)	<0.001
≥2	647 (3.0)	344 (2.9)	303 (3.1)	
Number of medical diagnoses of chronic conditions				
0	1,377 (6.3)	603 (5.0)	774 (7.9)	
1–2	5,269 (24.1)	2,395 (19.8)	2,874 (29.5)	<0.001
≥3	15,195 (69.6)	9,098 (75.2)	6,097 (62.6)	

PT: professional training.

### 3.2 Prevalence and trends of polypharmacy in adults ≥65 years old by sex.

The prevalence of polypharmacy was 23.2% (*n* = 5,077). The prevalence in women was higher (28.1%) than in men (17.2%) (*p* < 0.001).

**FIGURE 1:**
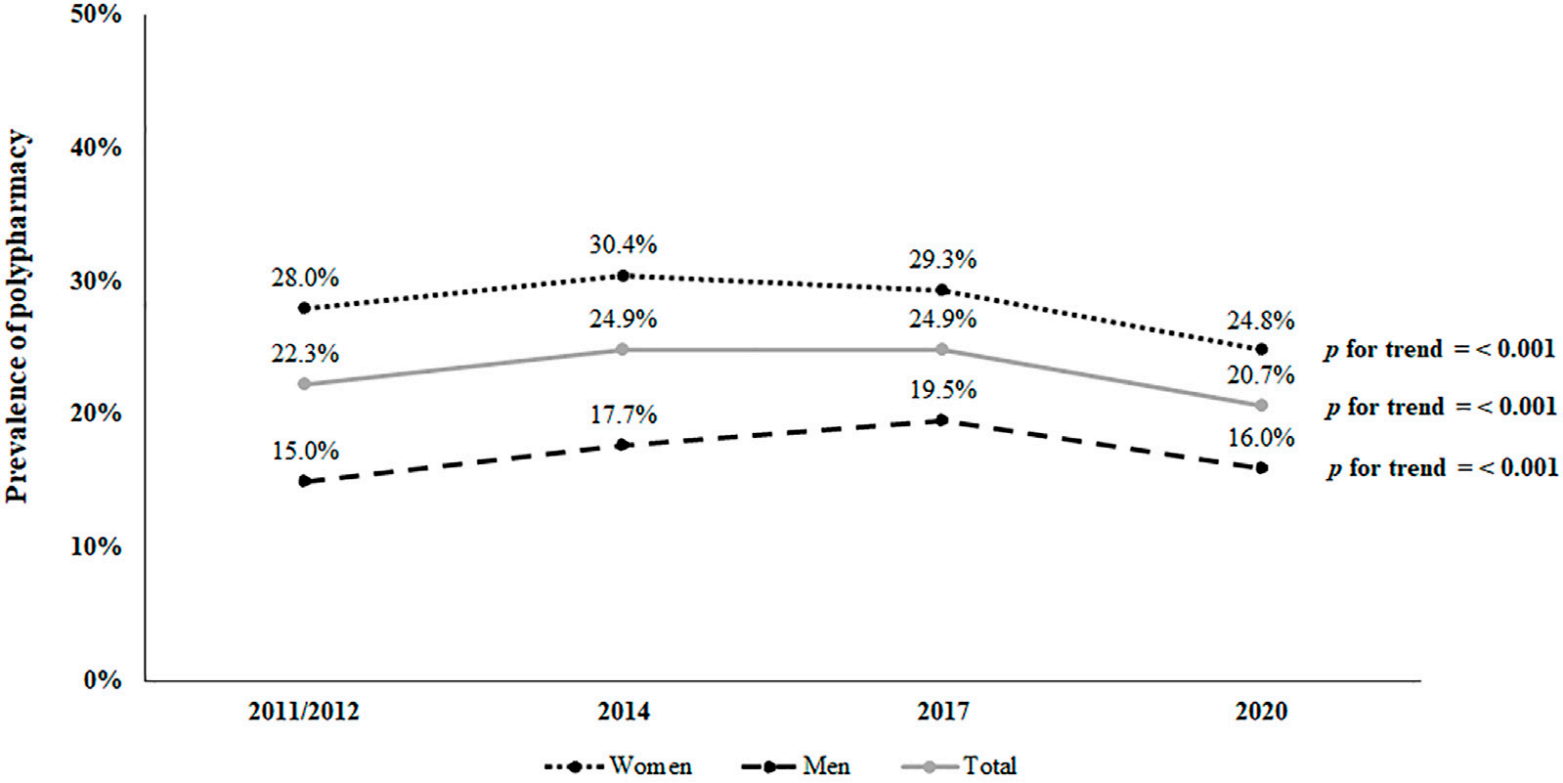
Distribution of prevalence of polypharmacy among elderly people from 2011/12 to 2020 (*n* = 5,077).

Figure 1 illustrates the distribution of the prevalence of polypharmacy between men and women over the study period (2011/12–2020). In general, the prevalence of polypharmacy decreased from the year 2011/2012 to the year 2020 (*p* < 0.001). The prevalence of polypharmacy in women decreased from 2011/2012 to 2020 (*p* < 0.001), with 2014 being the year with the highest prevalence (30.4%). In turn, the prevalence of polypharmacy in men increased over the years of the study (*p* < 0.001); even so, a decrease was found from the highest prevalence in 2017 (19.5%) to 2020 (16.0%) (Figure 1).

### 3.3 Medicine use in adults with polypharmacy ≥65 years old by sex

**FIGURE 2:**
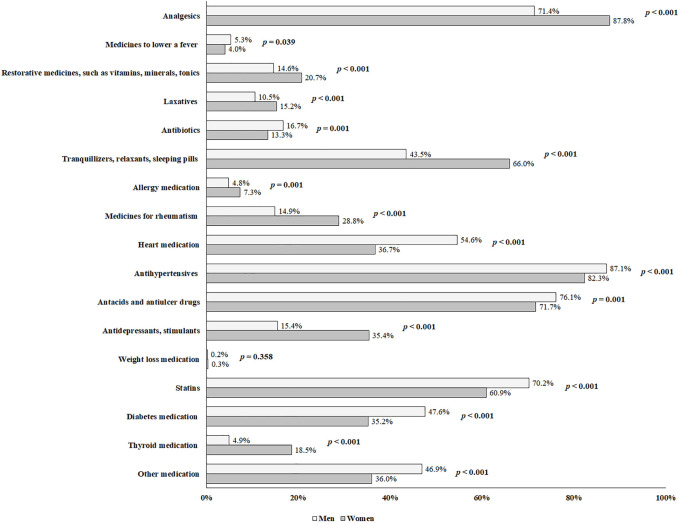
Distribution of medicine use in adults with polypharmacy ≥65 years old between men and women (*n* = 5,077).

**FIGURE 3:**
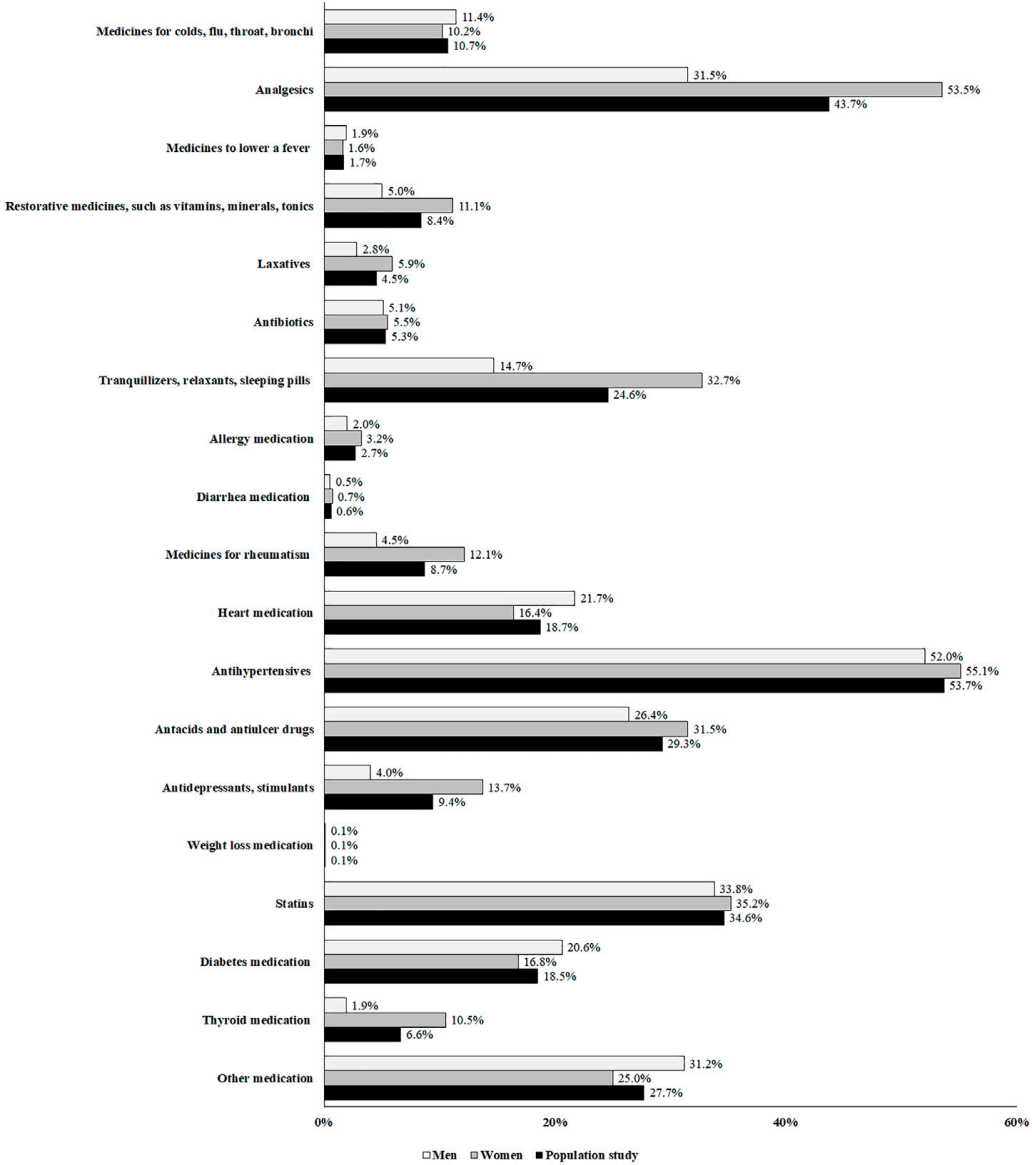
Distribution of medicine use in adults ≥65 years in the population study (*N* = 21,841).

The mean number of medicines consumed by all participants was 2.98 ± 2.15 (women: 3.30 ± 2.24; men: 2.59 ± 1.95: *p* < 0.001). The most commonly consumed medicines were analgesics (women: 87.8%; men: 71.4%; *p* < 0.001), followed by antihypertensives (women: 82.3%; men: 87.1%; *p* = 0.002), antacids and antiulcer drugs (women: 71.7%; men: 76.1%; *p* = 0.005), statins (women: 60.9%; men: 70.2%; *p* < 0.001), together with tranquillizers, relaxants and sleeping pills (women: 66.0%; men: 43.5%; *p* < 0.001) (Figure 2). Moreover, the consumed medicines in the population study were shown in the Figure 3.

### 3.4 Association between polypharmacy and sociodemographic, health-related variables and use of clinical care services in adults ≥65 years old by sex

Table 2 shows the crude and adjusted ORs allowing for the identification of determinants of polypharmacy in adults ≥65 years old by sex.

**TABLE 2:** Logistic regression analysis for determinants of polypharmacy in adults ≥65 years old by sex.

		Men (*n* = 9,745)			Women (*n* = 12,096)	
	OR (CI 95%)	ORa* (CI 95%)	*p*-value	OR (CI 95%)	ORa* (CI 95%)	*p*-value
Total						
Age intervals						
65–74 years	Reference	Reference		Reference		
75–84 years	1.81 (1.61–2.03)	1.19 (1.04–1.35)	0.01	1.72 (1.57–1.88)		
≥85 years	1.99 (1.67–2.34)	1.07 (0.89–1.30)	0.46	1.82 (1.63–2.04)		
Marital status						
Single	Reference			Reference		
Married	1.15 (0.96–1.40)			1.35 (1.13–1.61)		
Widowed	1.45 (1.17–1.80)			2.00 (1.69–2.39)		
Separated or divorced	0.92 (0.68–1.26)			1.10 (0.83–1.45)		
Social class						
Social classes I and II	0.67 (0.57–0.79)			0.55 (0.48–0.62)		
Social classes III and IV	0.88 (0.78–0.99)			0.78 (0.72–0.86)		
Social classes V and VI	Reference			Reference		
Educational level						
Without studies	Reference			Reference		
Primary	0.68 (0.60–0.78)			0.60 (0.54–0.66)		
Secondary or PT	0.50 (0.43–0.58)			0.47 (0.42–0.52)		
University	0.49 (0.40–0.60)			0.30 (0.25–0.37)		
Nationality						
Spanish	Reference			Reference		
Foreigner	0.64 (0.38–1.06)			0.40 (0.26–0.63)		
Size of town						
Rural	0.91 (0.81–1.02)			0.91 (0.83–1.02)		
Urban	Reference			Reference		
Self-perceived state of health						
Very good	Reference	Reference		Reference	Reference	
Good	2.35 (1.51–3.65)	1.27 (0.80–2.009	0.31	2.69 (1.83–3.96)	1.44 (0.96–2.15)	0.08
Average	10.67 (6.93–16.43)	2.77 (1.76–4.36)	<0.001	13.10 (9.00–19.08)	3.52 (2.37–5.24)	<0.001
Bad	23.41 (15.06–36.40)	4.19 (2.61–6.73)	<0.001	31.68 (21.61–46.43)	6.08 (4.05–9.14)	<0.001
Very bad	38.54 (23.61–62.92)	6.26 (3.66–10.71)	<0.001	37.39 (24.88–56.19)	6.56 (4.22–10.18)	<0.001
Current smoker						
Yes	1.65 (1.38–1.97)	1.33 (1.09–1.62)	<0.01	1.56 (1.28–1.90)		
No	Reference	Reference		Reference		
Alcohol intake in the last year						
Yes	0.66 (0.59–0.73)			0.57 (0.52–0.62)	0.82 (0.74–0.91)	<0.001
No	Reference			Reference	Reference	
Body Mass Index						
Underweight	1.69 (0.88–3.23)	0.74 (0.35–1.55)	0.42	1.22 (0.86–1.74)	0.88 (0.59–1.32)	0.54
Normal weight	Reference	Reference		Reference	Reference	
Overweight	1.21 (1.06–1.39)	1.30 (1.11–1.51)	<0.01	1.48 (1.34–1.63)	1.24 (1.11–1.39)	<0.001
Obesity	1.70 (1.46–1.98)	1.43 (1.20–1.70)	<0.001	2.49 (2.24–2.77)	1.65 (1.46–1.86)	<0.001
Degree of limitation due to a health problem for at least 6 months						
Severely limited	9.08 (7.66–10.78)	2.40 (1.92–3.00)	<0.001	8.79 (7.73–9.99)	2.37 (2.02–2.78)	<0.001
Limited but not severely	4.82 (4.26–5.45)	2.04 (1.77–2.35)	<0.001	4.79 (4.34–5.29)	1.91 (1.71–2.14)	<0.001
Not at all limited	Reference	Reference		Reference	Reference	
Visits to the family doctor in the preceding 4 weeks						
0	Reference	Reference		Reference	Reference	
1	1.87 (1.67–2.10)	1.38 (1.21–1.57)	<0.001	1.65 (1.51–1.80)	1.25 (1.13–1.38)	<0.001
≥2	4.43 (3.73–5.26)	2.27 (1.86–2.76)	<0.001	2.98 (2.61–3.42)	1.58 (1.35–1.84)	<0.001
Hospitalization during the past 12 months						
0	Reference	Reference		Reference	Reference	
1	2.17 (1.88–2.52)	1.22 (1.03–1.44)	0.02	1.93 (1.70–2.19)	1.21 (1.04–1.40)	0.01
≥2	3.26 (2.55–4.14)	1.28 (1.09–1.70)	0.03	3.44 (2.77–4.27)	1.67 (1.30–2.14)	<0.001
Number of medical diagnoses of chronic conditions						
0	Reference	Reference		Reference	Reference	
1–2	7.67 (1.87–21.50)	5.08 (1.23–6.96)	0.03	4.85 (1.17–20.14)	3.11 (0.74–4.98)	0.12
≥3	10.46 (8.68–12.78)	7.69 (5.31–9.30)	<0.001	7.30 (3.96–8.91)	4.23 (/3.13–8.51)	<0.001

PT: professional training; OR, odds ratio; *ORa, odds ratio adjusted for all sociodemographic variables, health-related determinants and use of clinical care services whose *p-*value ≤0.15; CI 95%, 95% Confidence Interval. Hosmer-Lemeshow test for men χ^2^ = 3.25, *p*-value = 0.92; Nagelkerke’s *R* ^2^ for men = 0.35; *p*-value <0.001. Hosmer-Lemeshow test for women χ^2^ = 2.50, *p*-value = 0.96; Nagelkerke’s *R* ^2^ for women = 0.37; *p*-value <0.001.

Missing data in women: Marital status: 28 (0.2%); Social class: 1,403 (8.8%); Nationality: 141 (0.9%); Current smoker: 20 (0.1%); Alcohol intake in the last year: 8 (0.1%); Body Mass Index (BMI): 2,414 (15.2%); Degree of limitation due to a health problem for at least 6 months: 2 (<0.1%); Number of medical diagnoses of chronic conditions: 82 (0.5%); Polypharmacy: 74 (0.5%).

Missing data in men: Marital status: 11 (0.1%); Social class: 97 (0.9%); Nationality: 58 (0.5%); Current smoker: 29 (0.3%); Alcohol intake in the last year: 16 (0.1%); Body Mass Index (BMI): 738 (6.9%); Degree of limitation due to a health problem for at least 6 months: 4 (<0.1%); Number of medical diagnoses of chronic conditions: 45 (0.4%); Polypharmacy: 44 (0.4%).

In men and women, the probability of polypharmacy was higher among those who perceived their state of health average, bad and very bad (women: OR = 3.52; OR = 6.08; OR = 6.56, respectively; *p* < 0.001; men: OR = 2.77; OR = 4.19; OR = 6.26, respectively; *p* < 0.001) and those who were overweight and obese (women: OR = 1.24; OR = 1.65, respectively; *p* < 0.001; men: OR = 1.30; OR = 1.43; *p* < 0.01, *p* < 0.001, respectively). Additionally, other positive predictors were being severely limited and limited (but not severely) due to a health problem for at least 6 months (women: OR = 2.37; OR = 1.91, respectively; *p* < 0.001; OR = 2.40; OR = 2.04, respectively; *p* < 0.001). The probability of polypharmacy was higher in those who had visited the family doctor in the preceding 4 weeks once and, at least, twice (women: OR = 1.25; OR = 1.58, respectively; *p* < 0.001; men: OR = 1.38; OR = 2.27, respectively; *p* < 0.001) and those who had been hospitalized during the past 12 months once and, at least, twice (women: OR = 1.21; OR = 1.67, respectively; *p* < 0.001; men: OR = 1.22; OR = 1.28, respectively; *p* < 0.01). In addition, having ≥ three chronic conditions (women: OR = 4.23; *p* < 0.001; men: OR = 7.69; *p* < 0.001) was associated with a higher probability of polypharmacy.

In women exclusively, alcohol consumption in the last year (OR = 0.82; *p* < 0.01) was associated with a lower probability of polypharmacy. In contrast, positive predictors were seen exclusively in men: being 75–84 years old (OR = 1.19; *p* = 0.01) and being a current smoker (OR = 1.33; *p* < 0.01).

## 4 Discussion

### 4.1 Main findings

The overall prevalence of polypharmacy in adults ≥65 years old residing in Spain was 23.2%, being higher in women (28.1%) than men (17.2%). This overall prevalence is in line with the range of percentages found by other authors who used SNHS or EHIS in elderly people (Martin-Pérez et al., 2017; Carmona-Torres et al., 2018; Gutiérrez-Valencia et al., 2019), but lower when compared to other European countries (Herr et al., 2017; Morin et al., 2018). Recently, in some areas there has been a stabilization or a slight decrease in polypharmacy (Canadian Institute for Health Information CIHI, 2016; Kristensen et al., 2019). This is the case of the prevalence in our study, which decreased from 2011/2012 (22.3%) to the year 2020 (20.7%). This small decrease may be due to the fact that the clinical profile of elderly adults is complex and that clinical interventions are largely unsuccessful (Rankin et al., 2018). For example, during the COVID-19 pandemic (study year 2020), polypharmacy predisposes older people to an increased risk of severe COVID-19 infection and mortality (Iloanusi et al., 2021). For that reason, this decrease could be attributed to arguments in favor of deprescribing, particularly among older COVID-19 patients with polypharmacy due to the incidence of drug interactions in patients increases with treatments to control COVID-19 disease (Cattaneo et al., 2020; Sürmelioğlu et al., 2021). Other possible explanations for the decrease in polypharmacy observed in 2020 include a complex interaction between effects related to the COVID-19 shutdown, such as the ban on non-urgent health services, individuals’ fear of contracting the virus, or individuals’ attempts to keep healthcare services from becoming overwhelmed (Barten et al., 2022). According to Rachamin et al. (Rachamin et al., 2021), patients in high-risk groups, such as older adults, may have been more concerned about contracting COVID-19 at the family doctor’s office. In addition, the prevalence of polypharmacy in men in our study increased over time, in contrast with the prevalence in women, which decreased. These downward trends in polypharmacy in elderly women may be due to the fact that more and more research has been published advocating a reduction in potentially dangerous and high-risk medications for women, especially due to their interactions and properties, compared to men (Canadian Institute for Health Information CIHI, 2016).

Analgesics, antihypertensives, antacids and antiulcer drugs, statins and tranquillizers, relaxants or sleeping pills were, in that order, the most commonly consumed medicines in the study participants, in line with other studies (Masnoon et al., 2017). Elderly women also used more analgesics and tranquillizers, relaxants or sleeping pills to alleviate painful diseases, whereas men used more antihypertensives, antacids and antiulcer drugs for hypertension and cardiovascular diseases (Collerton et al., 2009).

In men and women, our findings revealed that polypharmacy is linked to a several health-related variables as positive predictors, such as average, bad and very bad self-perceived state of health, being overweight and obesity, being severely limited and limited (but not severely) due to a health problem for at least 6 months and having ≥ three chronic conditions. Regarding the first factor, several studies found a high prevalence of polypharmacy among elderly adults who rated their health as poor or very poor (Gutiérrez-Valencia et al., 2019). In line with several studies (Carmona-Torres et al., 2018; Slater et al., 2018), polypharmacy was linked in our study to overweight and obesity, probably due to the wide array of comorbidities associated with higher BMI (Xia et al., 2021). Similarly, most elderly people have one or more limitations due to a health problem as they age and their health condition deteriorates, which means they need more medication (Kim et al., 2018). In this latter case, we observed a significant association between the number of chronic conditions and polypharmacy. In fact, polypharmacy is mainly a consequence of having numerous chronic diseases (Khezrian et al., 2020). Finally, polypharmacy is linked to the number of visits to the family doctor in the preceding 4 weeks and the number of hospitalizations during the past 12 months, as risk factors, in accordance with other studies (Resnick et al., 2018; O’Regan et al., 2022).

In elderly women, alcohol consumption is a negative predictor of polypharmacy. Similar findings (Slater et al., 2018) discovered a statistically significant inverse relationship between alcohol consumption and polypharmacy. Our findings support the positive outcomes of education programs aimed at older people to minimize risky medication and alcohol use habits (Benza et al., 2010). The association between alcoholic beverages and cardiovascular disease, among other things, has been defined as a U-curve (San José et al., 1999)–moderate intake is associated with a lower likelihood of having cardiovascular diseases when compared to abstention or persons who consume excessively. Pedroso-Remelhe et al. (Pedroso-Remelhe et al., 2022) explained the association observed in the present study—those with moderate consumption may have fewer comorbidities or a better general health state, necessitating fewer medicines and/or supplements. For its part, Antonelli-Incalzi et al. (Antonelli Incalzi et al., 2005) attribute this result to a bias caused by the fact that individuals in better health are less motivated to change bad habits. Nevertheless, this result needs to be explored.

Former smokers were found to have a higher risk of polypharmacy than non-smokers (Castioni et al., 2017). Our results showed that polypharmacy is associated with being a current smoker in elderly men, and it is clear that polypharmacy is related to being 75–84 years old in elderly men, which is supported by Qato et al. (Qato et al., 2008).

### 4.2 Strengths and limitations

One of the study’s major strengths is the use of a large, nationally representative sample of elderly people, which contributes to the generalizability of the findings. Another advantage is the fact that the methodology was used consistently over the time period, as well as the large number of socio-demographic and health-related determinants and clinical care service use variables collected. Nonetheless, there are several study limitations. First, because this is a cross-sectional study, the causality of the associations cannot be determined. The second limitation is that information gleaned from an interview may be subject to memory or social desirability biases. Third, the surveys did not include all types of medications, nor did they quantify the number of medications of the same type. Fourth, both SNHS and EHIS considered medicines that were not prescribed by a healthcare professional. Fifth, BMI was calculated using the subjects’ self-reported heights and weights, which may or may not be accurate. Finally, the SNHS and EHIS surveys were conducted with various samples.

### 4.3 Implications for research and practice

Regarding medicine use in adults with polypharmacy, more evidence about which drugs frequently contribute to polypharmacy may help to inform effective interventions to reduce polypharmacy in elderly adults (Wastesson et al., 2018). To avoid the health problems associated with polypharmacy, health professionals must continuously reassess the medication regime and current clinical status of elderly people48. The findings of this study should be considered by health authorities when developing or improving health guidelines and strategies for promoting the proper use of medication, particularly in the elderly population by sex. Lastly, it would be of great interest to carry out further research into the potentially significant influence of gender on polypharmacy and its associated factors in order to improve the safety of using medication in the elderly population.

## 5 Conclusion

In Spain, polypharmacy has a prevalence of 23.2%, with women accounting for 28.1% and men accounting for 17.2% of the total. The prevalence of polypharmacy decreased among elderly women from 2011/2012 to 2020, while it has increased over time among elderly men. Analgesics and tranquillizers, relaxants or sleeping pills are the most commonly used medicines in elderly women, while antihypertensives, antacids and antiulcer drugs, and statins are the most frequently used in elderly men. In men and women, average, bad, and very bad self-perceived state of health, people with overweight and obesity, being severely limited and limited (but not severely) due to a health problem, having ≥ three chronic conditions, visits to the family doctor and hospitalization are all positive predictors of polypharmacy. Alcohol consumption is a negative predictor among elderly women, whereas in elderly men being 75–84 years old, being a current smoker and having 1–2 chronic conditions are positive predictors.

## Data Availability

Publicly available datasets were analyzed in this study. This data can be found here: https://www.sanidad.gob.es/estadisticas/microdatos.do.
